# Pregnancy after kidney transplantation: global insights based on registry data from three continents

**DOI:** 10.1007/s40620-025-02451-x

**Published:** 2025-11-22

**Authors:** Styliani Giapoutzidou, Erandi Hewawasam, Margriet E. Gosselink, A. Titia Lely, Michael J. Moritz, Serban Constantinescu, Lisa Coscia, Shilpanjali Jesudason, Margriet F. C. de Jong

**Affiliations:** 1 Department of Nephrology, University Medical Center Groningen, Groningen, Netherlands; 2 Australia and New Zealand Dialysis and Transplant Registry, South Australian Health and Medical Research Institute, Adelaide, Australia; 3 Faculty of Health and Medical Sciences, University of Adelaide, Adelaide, Australia; 4 Department of Obstetrics and Gynecology, University Medical Center Utrecht, Utrecht, Netherlands; 5 Transplant Pregnancy Registry International, Gift of Life Institute, Philadelphia, PA USA; 6 Medicine, Lewis Katz School of Medicine at Temple University, Philadelphia, PA USA; 7 Central Northern Adelaide Renal and Transplantation Services, Royal Adelaide Hospital, Adelaide, Australia

**Keywords:** Registry, Methodology, Pregnancy, Transplantation, Kidney disease

## Abstract

**Background:**

Lack of data regarding pregnancy post-kidney transplantation challenges clinicians who are faced with complex, high-risk cases. Aiming at tackling knowledge gaps and limited cross-cultural data on pregnancy in kidney transplant recipients (KTRs), we compared the methodologies and pregnancy outcomes of three registries based in three continents.

**Methods:**

Data were gathered from reports and publications of the Pregnancy After Renal Transplantation OUTcomes registry (PARTOUT, Netherlands), the Australia and New Zealand Dialysis and Transplant Registry (ANZDATA), and the Transplant Pregnancy Registry International (TPRI, United States of America and international). We targeted the similarities and differences among the registries to understand methodological variations.

**Results:**

The registries utilized distinct approaches regarding data collection which influence data interpretation. PARTOUT conducted a retrospective analysis of all Dutch pregnant KTRs between 1971 and 2017. ANZDATA includes annual surveys on all KTR parenthood events since 1968. TPRI offers international coverage and includes pregnant KTRs voluntarily registered since 1991. Despite methodological differences, preeclampsia, preterm birth and low birth weight were common pregnancy complications, and outcomes were mostly comparable among the registries.

**Conclusions:**

Despite differences in case capture, the three registries reported similar pregnancy and newborn outcomes, confirming that pregnancy in KTRs can be successful with careful monitoring across varying populations. Identifying the strengths and weaknesses of each registry can contribute to improved methodologies for global data collection and lower missing data rates. Although managing large databases may be challenging, aligning data across countries could lead to meaningful data pooling, while identifying drivers of outcomes across subpopulations.

**Graphical abstract:**

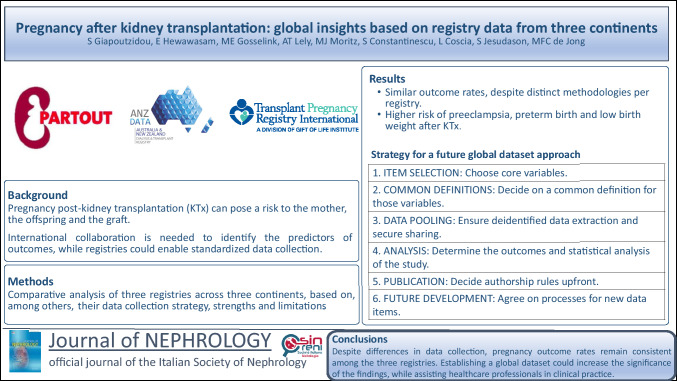

**Supplementary Information:**

The online version contains supplementary material available at 10.1007/s40620-025-02451-x.

## Introduction

Kidney transplantation (KTx) aims to enhance the duration and quality of life of patients with kidney failure, and for some recipients, particularly young women, this could include pregnancy and childbearing [1]. KTx improves fertility [2], which declines in patients with kidney failure [3]. However, pregnancy can pose increased risks to the recipient, the graft, and the fetus. With increasing numbers of post-KTx pregnancies [4], there is a growing need to ensure maternal and neonatal safety by identifying the most common challenges in clinical practice.

Three pregnancy registries that have informed the field are the Pregnancy After Renal Transplantation OUTcomes (PARTOUT) in the Netherlands, the Australia and New Zealand Dialysis and Transplant Registry (ANZDATA), and the Transplant Pregnancy Registry International (TPRI) in the United States of America.

Overall, pregnancy post-KTx remains relatively uncommon, creating the need for international collaboration to gain sufficient power for meaningful examination of outcomes and trends. Registries enable standardized data collection across centers, and contribute to improved data capture strategies that could mitigate reporting bias and missing data rates. Therefore, the aim of this study was to compare the methodology of these three registries, while identifying their focus to facilitate future research collaboration with shareable datasets.

## Methods

We compared pregnancy data from three existing registries: PARTOUT, ANZDATA, and TPRI, each of which included women who conceived post-KTx. Data were gathered based on the ANZDATA and TPRI annual reports up until 2022 [5–7] and publications from all three registries [4, 8, 9]. The current study aimed to evaluate their methodologies, strengths and limitations rather than to generate new data or merge the datasets. The comparison of the datasets was based on data collection strategy, duration, number of participants, inclusion and exclusion criteria, strengths, limitations, and funding. Maternal, neonatal, graft outcomes and other clinical findings were also reported. Since no primary data were generated, ethics approval was not required, and each registry had already been approved separately.

## Results

The main characteristics of each registry are summarized below and listed in Table 1.

**Table 1:** Descriptive data of the three datasets

	PARTOUT [8]	ANZDATA [7]	TPRI [5]
Duration^a^	1971–2017	1968–2021	1991–2022
Current status	Data collection stopped in 2017. PARTOUT has now evolved into the Pregnancy, Genetics & Nephrology network (PREGeNEPH).	Ongoing	Ongoing
Parenthood events	301	2643	3460
Maternal events (*n*,%)	301 (100)	1135 (43)	2455 (71)
Paternal events (*n*, %)	Not included (0)	1508 (57)	1005 (29)
Female kidney transplant recipients (KTRs)	202	757	1367
Inclusion criteria	Pregnancy in female KTRs, 288 singleton gestations > 20 weeks.	Females that conceived or became pregnant while on kidney replacement therapy (KRT), males that fathered a child post-KRT.	Female transplant recipients that were or are currently pregnant, males that have fathered a child after transplantation, all solid organs included.
Exclusion criteria	13 twin pregnancies	Those who received dialysis for less than 100 days.	Patients are excluded if they cannot communicate in English or provide a translator.
Other organ transplantations included	No	Yes	Yes
Data collection methodology	Aimed for nationwide inclusion, by systematic patient search via the National Organ Transplant Registry and via consulting transplant nephrologists and obstetricians in participating centers. A dedicated research team gathered data from medical charts of patients who had regular check-ups at the university medical centers during pregnancy.	Voluntary participation that includes paper surveys and online forms with both obligatory and non-mandatory fields.	Registration via health provider or self-enrollment. Interviews with the recipient when enrolled, after delivery and every two years (patient reported outcomes).
Completeness	Composite adverse pregnancy outcome (cAPO): > 80% cAPO was defined as at least one of the following: birth before week 37 of the gestation, low birth weight (< 2500 g), > 15% increase in serum creatinine (SCr) in the last trimester in comparison to prepartum SCr, > 160 mmHg systolic blood pressure and/or > 110 mmHg diastolic blood pressure in the last trimester.	Core data (pregnancy and neonatal outcomes, medical conditions acquired during gestation): > 85% Granular data items added after 2018 (dialysis data, labor, delivery, medications used during pregnancy): < 85%	Not available.
Lower completeness/missing data rates	Antihypertensives: 20%, Apgar scores: 35%, Neonatal Intensive Care Unit (NICU) admission: 30%	Granular dialysis-related data. Occurrence of preeclampsia.	Not available.
Strengths	Consecutive nationwide coverage of pregnancy in KTRs, clinically relevant questions answered, data published for counseling and monitoring, access to detailed data.	Bi-national, founded the earliest among the three, reported data completeness rates. Data linkage to perinatal registries has allowed expansion of the data items.	International approach and follow-up to determine long-term effects. Immunosuppressant exposure data.
Limitations	Retrospective data collection by different members of the research team, paper to digital form, long inclusion time, different guidelines and organization of care during the years.	Limited obstetric and delivery data, blank fields, lack of detailed data, reporting bias. Data linkage to perinatal registries has allowed some of these limitations to be overcome.	Skewed towards North American participants, selection and recall bias possible.
Evolution over time	After 2002 there were more pregnancies after a living donor kidney transplantion [4]. After 2002, there was an increasing number of mothers with pre-existing hypertension and higher incidence of preeclampsia [4].	First cases from the 1970s. A specific survey was introduced in 2001. Data expansion occurred in 2018. Since 2018: pregnancy outcome is mandatory, gestational age is 95% complete, questions with the unknown option have higher completeness, complex clinical data have lower completeness.	In 2010, added prospective reporting, allowing participation of pregnant women whose pregnancy outcome is unknown. Subsequent comparison has shown no difference in pregnancy outcomes between those prospectively vs retrospectively reported (manuscript in preparation). In 2016 it expanded to enroll participants worldwide.
Funding	None, indirect sponsoring from Nierstichting.	- Australian Organ and Tissue Donation and Transplantation Authority - NZ Ministry of Health - Kidney Health Australia - Australian and New Zealand Society of Nephrology (ANZSN)	- The Transplant Foundation of Gift of Life. - Previously, grants from immunosuppressant producers and companies involved in the transplant field. - Private donations.
How to Contact	-	https://www.anzdata.org.au/anzdata/	https://www.transplantpregnancyregistry.org/

^a^Duration of the annual reports from which the data depicted in this table were obtained. Duration of each registry is the same, except for ANZDATA and TPRI that still actively collect data to this day

### PARTOUT

**Fig. 1:**
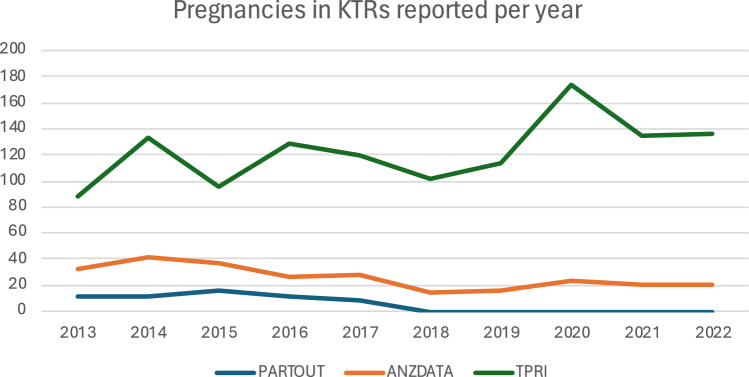
Number of pregnancies in KTRs reported annually by each registry

PARTOUT was a cohort study established in 2017 involving all eight kidney transplant centers in the Netherlands. It included all pregnant KTRs in the country from 1971 until 2017, when the data collection was completed. Participants were identified via the National Organ Transplant Registry, where all Dutch transplant patients are registered. Further consultation with obstetricians and transplant nephrologists in the participating centers contributed to finalizing participant inclusion. Data were collected from medical charts at the transplant centers by dedicated members of the research team. The highest number of pregnancies reported in a year was 16 in 2015, while the lowest was 8 in 2017 (Fig. 1). In 2024, the PARTOUT network evolved into the Pregnancy, Genetics & Nephrology (PREGeNEPH) registry that actively collects data prospectively on pregnancy in KTRs and chronic kidney disease patients.

#### Strengths and limitations of PARTOUT

PARTOUT included all pregnancies post-KTx in the country, thus providing comprehensive national coverage. Moreover, the data were collected from medical charts, hence avoiding self-reported data and lowering selection bias. PARTOUT also conducted the largest study worldwide on how pregnancy post-KTx affects long-term estimated glomerular filtration rate (eGFR) [9].

Limitations were that data collection came to an end on December 31, 2017 and only retrospective data were collected. The data were gathered from a long time interval and missing data could not be retrieved. Relying on input from healthcare professionals to determine patient inclusion and excluding pregnancies from the analysis because of missing data could have introduced bias.

### ANZDATA

ANZDATA is a bi-national, ongoing, prospective registry that annually gathers clinical data from dialysis and transplant units in Australia and New Zealand [6, 7]. ANZDATA captures maternal and paternal events reported while receiving kidney replacement therapy ([KRT]; chronic dialysis or kidney transplantation). ANZDATA has 100% capture of KRT recipients. Parenthood events, reported via the treating transplant unit, have been captured since 1968, but a parenthood form was introduced in 2001 to formalize this data collection process. Paper surveys and online forms with obligatory fields have been utilized for data collection. During the last ten years the highest number of annually reported pregnancies post-KTx was 30 pregnancies in 2014, while the lowest was 15 in both 2016 and 2018 (Fig. 1).

#### Strengths and limitations of ANZDATA

ANZDATA is the oldest among the three registries and started collecting data prospectively. Additionally, registry staff monitor data completeness across various variables, further adjusting the data collection via liaison with treating units.

Regarding the limitations of the registry, surveys are completed on paper or online, though not all items are mandatory to enter, and fields may be left blank. The data collectors are renal unit staff who may not have the entire pregnancy record available if obstetric care occurred at a different site, resulting in missing data [7]. Case capture has been previously validated against birth registries and is higher than 75% [7].

### TPRI

TPRI is a voluntary pregnancy registry that has been actively collecting data since 1991 and includes pregnancies from 1968 onward. In 2010, TPRI began collecting data prospectively in addition to retrospective data collection. Solid organ transplant recipients who have had or fathered a pregnancy are included. The highest number of pregnancies post-KTx included per year was 150 pregnancies in 2020 (Fig. 1), when the COVID-19 pandemic began. Registration takes place via a health provider or self-enrollment, while interviews take place at enrollment, post-delivery, and then every one to two years as long as recipients can be reached. In 2016, TPRI expanded to include participants from all countries [5]. Participants are excluded if they cannot communicate in English or provide a translator. There are 14 Australian KTRs with 24 pregnancies and 1 Dutch KTR with 4 pregnancies included in TPRI.

#### Strengths and limitations of TPRI

Strengths of TPRI include long-term follow-up and global inclusion. Data collection on non-KTR pregnancies after solid organ transplantation leads to inclusion of different end-stage organ failure groups that could be at higher risk of adverse pregnancy or graft outcomes than KTRs.

However, limitations include its voluntary nature, which makes it susceptible to selection bias. Furthermore, the number of participants from countries outside the USA is low, incompletely filling the need for participants from different countries for more inclusive data capture. Lastly, when data are collected from participants retrospectively, recall bias can be introduced.

### Outcomes

Pregnancy, neonatal, and graft outcomes are listed in Table S1. Regarding pregnancy outcomes, PARTOUT includes live births only past the 20th gestational week. ANZDATA and TPRI report miscarriages before the 20th gestational week. All three registries report stillbirth defined as after the 20th gestational week. Ectopic pregnancies are reported only by TPRI, and termination rates by ANZDATA and TPRI. Reported live birth rates among PARTOUT, ANZDATA, and TPRI ranged between 75–93%. Mean gestational age was approximately 35 weeks for all registries, while mean birth weight was 2383, 2360 (median), and 2551 g for PARTOUT, ANZDATA, and TPRI, respectively. Approximately one third of the participants had preeclampsia across all registries. Graft loss was reported in 23% of the PARTOUT recipients at a median time of 6.44 years post-delivery. In ANZDATA, 27% of KTRs experienced graft loss with a median follow-up of 8.08 years postpartum, while 33% of TPRI participants had graft loss at their last follow-up 14.4 years post-delivery.

## Discussion

Our study compared three large registries collecting post-KTx pregnancy data. The registries were influenced by the investigators’ interests and limited by regulatory restrictions, therefore, developing distinct methodological approaches. Pregnancy outcomes appear consistent across the registries, despite the differences in population size and methodology. Preeclampsia, preterm birth, and low birth weight are some of the most common adverse maternal and neonatal outcomes in KTRs (Table S1 and Table S2). Consequently, pregnancy post-KTx is classified as high-risk. However, the risk can be moderated through advance planning and close monitoring. Although pre-pregnancy kidney function and blood pressure are the strongest predictors of a successful pregnancy outcome (Table S2), healthcare professionals should remain vigilant regardless of a lower-risk prediction.

The current study compared data from diverse populations. Different populations have distinct demographics which could be used to navigate research towards what is, for example, prevalent in a specific group. Each registry has accumulated different numbers of pregnancies and has different inherent biases that affect the statistical analysis and generalizability of the findings. Similar outcomes among the registries contributed to widely applicable conclusions on pregnancies post-KTx.

What is the next step? Establishing global datasets with increased data acquisition should enable improved identification of predictors of pregnancy and newborn complications and should include data from populations potentially under-represented in the existing registries. A step-by-step strategy for the future is presented in Table 2. It would likely shed more light on pregnancy post-KTx and establish a path for similar international initiatives, while tackling challenges in clinical practice.

**Table 2:** Strategy for a future global dataset approach

1. ITEM SELECTION: Choose core variables based on importance, availability and reliability. Items should be relevant, available and reliable in each contributing jurisdiction.
2. COMMON DEFINITIONS: Compare the definitions of those variables in each registry and decide on a common definition.
3. DATA POOLING: Ensure that the data can be shared safely taking into account regulations and ethics. Develop protocols for deidentified data extraction and secure sharing, either at individual level or pooled data.
4. ANALYSIS: Collectively determine the research question, aims, primary and secondary outcomes of the study and what type of statistical analysis will be performed. Compare findings and trends of the merged data with the ones from each registry prior to merging.
5. PUBLICATION: Decide authorship rules upfront and promote inclusive authorship for all contributors.
6. FUTURE DEVELOPMENT: Agree on processes for new data items considering sustainability and further aims.

## Conclusion

To conclude, pregnancy outcomes were similar among three registries, PARTOUT, ANZDATA and TPRI, despite variations in data collection. Intercontinental collaboration is essential to promote the development of global datasets, harmonize methodologies, improve data quality, and provide reliable information to support healthcare professionals in counseling kidney transplant recipients regarding pregnancy.

## Supplementary Information

Below is the link to the electronic supplementary material.

Supplementary file1 (DOCX 45 KB)

## Data Availability

The data presented in this study are available from PARTOUT, ANZDATA and TPRI. The authors do not own the data. Further information can be found online at https://www.anzdata.org.au/anzdata/ and https://www.transplantpregnancyregistry.org/.

## Abbreviations

ANZDATA: Australia and New Zealand Dialysis and Transplant Registry
eGFR: Estimated glomerular filtration rate
KTx: Kidney transplantation
KTRs: Kidney transplant recipients
LBW: Low birth weight
MPA: Mycophenolic acid
NICU: Neonatal intensive care unit
PARTOUT: Pregnancy After Renal Transplantation OUTcomes
PREGeNEPH: Pregnancy, Genetics & Nephrology
KRT: Kidney replacement therapy
SCr: Serum creatinine
TPRI: Transplant Pregnancy Registry International
