# Supplementary material for: Pregnancy after kidney transplantation: global insights based on registry data from three continents
Source: J Nephrol. 2025 Nov 22;38(9):2969–76. doi: 10.1007/s40620-025-02451-x (PMC12711958; doi:10.1007/s40620-025-02451-x)
Supplement: Supplementary file 1 — Supplementary file1 (DOCX 45 KB) [file 40620_2025_2451_MOESM1_ESM.docx]

**Supplementary Information**

**Pregnancy after kidney transplantation: global insights based on registry data from three continents**

Journal of Nephrology

Styliani Giapoutzidou BSc^1^, Erandi Hewawasam BMedSci PhD^2,3^, Margriet E. Gosselink MD PhD^4^, A. Titia Lely MD PhD^4^, Michael J. Moritz, MD^5^, Serban Constantinescu, MD, PhD^5,6^, Lisa Coscia* RN, BSN^5^, Shilpanjali Jesudason* MD PhD^2,3,7^, Margriet FC de Jong* MD PhD^1^.

*****shared last authors

**Affiliations**

1. Department of Nephrology, University Medical Center Groningen, Groningen, Netherlands

2. Australia and New Zealand Dialysis and Transplant Registry, South Australian Health and Medical Research Institute, Adelaide, Australia

3. Faculty of Health and Medical Sciences, University of Adelaide, Adelaide, Australia

4. Department of Obstetrics and Gynecology, University Medical Center Utrecht, Utrecht, Netherlands

5. Transplant Pregnancy Registry International, Gift of Life Institute, Philadelphia, PA, USA

6. Medicine, Lewis Katz School of Medicine at Temple University, Philadelphia, PA, USA

7. Central Northern Adelaide Renal and Transplantation Services, Royal Adelaide Hospital, Adelaide, Australia

**Corresponding author:**

Styliani Giapoutzidou

[s.giapoutzidou@umcg.nl](mailto:s.giapoutzidou@umcg.nl)

**Supplementary table S1** Pregnancy and transplantation outcomes for kidney transplant recipients

|  | PARTOUT | ANZDATA | TPRI [7] |
| --- | --- | --- | --- |
| **Epidemiology** |  |  |  |
| Median age at conception, yr | 31 [1] | 33 [3] | 30.4 (mean) |
| Median BMI at pregnancy | 24 [1] | 24.5 [4] | - |
| Race | 84% Caucasian [1] | 80% Caucasian [5] | ~ 70% Caucasian |
| Median transplant-conception interval, yr | 5 [1] | 5.3 [6] | 5.4 (mean) |
| **Pregnancy outcomes** |  |  |  |
| Live births | 93% [1] | 86% [3]  96.7% when ≥20 weeks of gestation [4] | 75% |
| Miscarriage | - | 8.5% [3] | 19% |
| Stillbirth | 7% [1] | 3.3% [4] | 2% |
| Ectopic pregnancies | - | - | 1% |
| Terminations | - | 5.6% [3] | 4% |
| Neonatal deaths | 3% [1] | 2.9% [4] | 1% |
| Mean gestational age, weeks | 35.6 [1] | 35 (median) [3] | 35.8 |
| Mean birth weight (g) | 2383 [1] | 2360 (median) [3] | 2551 |
| **Preconception** |  |  |  |
| Immunosuppression* | 87-91% Prednison  71-73% Aza  48-52% CNI (29% CsA, 23% Tac)  1% Other  during pregnancy [1] | 77-80% Prednisolone  64-74% Aza  21-33% MPA  91% CNI  (45-67% Tac  30-43% CsA)  1-4% Others  at conception [5] | Primary: 40% Tac,  38% CsA, 22% Other.  Overall (primary or secondary): 72% Aza,  8% MPA,  1% Sirolimus  during pregnancy |
| Preconception eGFR/creatinine | 61 mL/min/1.73 m^2^ mean eGFR [1]  117 μmol/L mean serum creatinine [1] | 54.1  mL/min/1.73 m^2^  (IQR: 44-65.4) median eGFR [6]  106 μmol/L  (IQR : 90-130) median serum creatinine [6] | - |
| **Maternal outcomes** |  |  |  |
| Postpartum eGFR/creatinine | 56.4 mL/min/1.73 m^2^ mean eGFR  (2.80 mL/min/1.73 m^2^ decrease in mean eGFR after the first pregnancy) [2] | 51.9  mL/min/1.73 m^2^  (IQR: 43.8-65.6) median eGFR [6]  110 μmol/L  (IQR: 90-130) median serum creatinine [6] | - |
| Preeclampsia | -34% [1]  -26% gestational hypertension [1] | 37% [5] | 30% |
| Gestational diabetes | - | 5.2% [4] | - |
| **KT outcome** |  |  |  |
| Long term graft outcome | 23% graft loss with a median post-delivery time of 6.44 years [1] | 27% graft loss with a median post-delivery time of 8.08 years [5] | 33% graft loss at a mean of 14.4 years post-delivery |

eGFR, estimated glomerular filtration rate

*azathioprine (Aza); calcineurin inhibitors (CNI); tacrolimus (Tac); cyclosporine or its modified form (CsA); mycophenolic acid (MPA)

**Supplementary Table S2** Summary of conclusions from registry pregnancy data analyses

|  | PARTOUT | ANZDATA | TPRI |
| --- | --- | --- | --- |
| Risk factors and possible predictors of outcomes | -Lower eGFR before pregnancy showed decreased pregnancy duration and lower birth weight [1].  -Blood pressure and serum creatinine could predict adverse pregnancy outcomes [1]. | -Preconception eGFR <45 ml/min/1.73 m^2^ and preconception SCr ≥1.24 mg/dL were linked to higher risk of adverse outcomes and graft failure [5]. | In unplanned pregnancies, there is an increased risk of termination, acute kidney rejection, and graft loss within 2 years of pregnancy [7]. |
| Pregnancy, neonatal and graft outcomes | -Relatively good pregnancy outcomes post-KTx: still, KTRs had a higher incidence of preterm births, LBW, small for gestational age compared to chronic kidney disease patients [1].  -KTRs who became pregnant before 1990 had a better eGFR following transplantation compared to those who became pregnant later [2].  -Pregnancy post-KTx can result in a slightly decreased eGFR [2]. | -Excellent live birth rates of ≥94% in females on kidney replacement therapy (KRT) whose pregnancy reached the 20^th^ gestational week [4].  -The preeclampsia rate among KTRs has nearly doubled in the last decade [5].  -KTRs who developed preeclampsia were at increased risk of adverse fetal outcomes [5].  -Preeclampsia did not impact graft outcomes [5].  -Preterm babies of females undergoing RRT had more than double the risk of adverse perinatal outcomes than other preterm babies [4]. | -Preterm delivery and fetal growth restriction have been observed but rather inconsistently when azathioprine is used [9].  -Acute rejection is associated  with higher creatinine levels, and preeclampsia is associated  with increased proteinuria [10].  -Trial of Labor vs a scheduled cesarean delivery was associated with improved neonatal outcomes among KTRs and with no increased severe maternal morbidity [11].  -Breastfeeding is safe for most KTRs after delivery [12]. |
| Immunosuppression | Calcineurin inhibitor use led to higher serum creatinine, more cases of lower birth weight, and increased need for antihypertensives [8]. | Increased exposure to calcineurin inhibitors in KTRs that had preeclampsia [5]. | -For women, fetal first trimester MPA product exposure results in miscarriage >40% (fetotoxicity) and birth defect incidence 18% with mycophenolate embryopathy among live births [9].  -No increase in birth defects or miscarriages reported with sirolimus pregnancy exposure. The miscarriage rate was 18% and the birth defects rate was 5.7% [7]. |

MPA, Mycophenolic acid

**References**

1. Gosselink ME, van Buren MC, Kooiman J, et al. A nationwide Dutch cohort study shows relatively good pregnancy outcomes after kidney transplantation and finds risk factors for adverse outcomes. *Kidney International*. 2022;102(4):866-875. doi:10.1016/j.kint.2022.06.006
2. van Buren MC, Gosselink M, Groen H, et al. Effect of pregnancy on eGFR after kidney transplantation: A national cohort study. *Transplantation*. 2022;106(6):1262-1270. doi:10.1097/tp.0000000000003932
3. Hewawasam E, Davies C, Jain A, McDonald S, Jesudason S. ANZDATA Special Report: Parenthood Survey 2022. Australia and New Zealand Dialysis and Transplant Registry, Adelaide, Australia. 2024. ISBN: 978-0-6453621-5-2. Available at: http://www.anzdata.org.au
4. Hewawasam E, Davies CE, Li Z, et al. Determinants of perinatal outcomes in dialyzed and transplanted women in Australia. *Kidney International Reports*. 2022;7(6):1318-1331. doi:10.1016/j.ekir.2022.03.015
5. Lu J, Hewawasam E, Davies CE, Clayton PA, McDonald SP, Jesudason S. Preeclampsia after kidney transplantation. *Clinical Journal of the American Society of Nephrology*. 2023;18(7):920-929. doi:10.2215/cjn.0000000000000155
6. Wyld ML, Clayton PA, Jesudason S, Chadban SJ, Alexander SI. Pregnancy outcomes for kidney transplant recipients. *American Journal of Transplantation*. 2013;13(12):3173-3182. doi:10.1111/ajt.12452
7. Transplant Pregnancy Registry International (TPRI) 2022 Annual Report, Gift of Life Institute, Philadelphia, PA 2023
8. Koenjer LM, Meinderts JR, Heijden OW, et al. Comparison of pregnancy outcomes in Dutch kidney recipients with and without calcineurin inhibitor exposure: A retrospective study. *Transplant International*. 2021;34(12):2669-2679. doi:10.1111/tri.14156
9. Coscia LA, Constantinescu S, Davison JM, Moritz MJ, Armenti VT. Immunosuppressive drugs and fetal outcome. *Best Practice & Research Clinical Obstetrics & Gynaecology*. 2014;28(8):1174-1187. doi:10.1016/j.bpobgyn.2014.07.020
10. Yin O, Kallapur A, Coscia L, Constantinescu S, Moritz M, Afshar Y. Differentiating acute rejection from preeclampsia after kidney transplantation. *Obstetrics & Gynecology*. 2021;137(6):1023-1031. doi:10.1097/aog.0000000000004389
11. Yin O, Kallapur A, Coscia L, et al. Mode of obstetric delivery in kidney and liver transplant recipients and associated maternal, neonatal, and graft morbidity during 5 decades of clinical practice. *JAMA Network Open*. 2021;4(10). doi:10.1001/jamanetworkopen.2021.27378
12. Constantinescu S, Pai A, Coscia LA, Davison JM, Moritz MJ, Armenti VT. Breast-feeding after transplantation. *Best Practice & Research Clinical Obstetrics & Gynaecology*. 2014;28(8):1163-1173. doi:10.1016/j.bpobgyn.2014.09.001
